# Genotyping-by-sequencing of pear (*Pyrus* spp.) accessions unravels novel patterns of genetic diversity and selection footprints

**DOI:** 10.1038/hortres.2017.15

**Published:** 2017-04-12

**Authors:** Satish Kumar, Chris Kirk, Cecilia Deng, Claudia Wiedow, Mareike Knaebel, Lester Brewer

**Affiliations:** 1 The New Zealand Institute for Plant & Food Research Limited, Hawke’s Bay Research Centre, Havelock North, New Zealand; 2 Palmerston North Research Centre, Palmerston North, New Zealand; 3 Mount Albert Research Centre, Auckland, New Zealand; 4 Motueka Research Centre, Motueka, New Zealand

## Abstract

Understanding of genetic diversity and marker-trait relationships in pears (*Pyrus* spp.) forms an important part of gene conservation and cultivar breeding. Accessions of Asian and European pear species, and interspecific hybrids were planted in a common garden experiment. Genotyping-by-sequencing (GBS) was used to genotype 214 accessions, which were also phenotyped for fruit quality traits. A combination of selection scans and association analyses were used to identify signatures of selection. Patterns of genetic diversity, population structure and introgression were also investigated. About 15 000 high-quality SNP markers were identified from the GBS data, of which 25% and 11% harboured private alleles for European and Asian species, respectively. Bayesian clustering analysis suggested negligible gene flow, resulting in highly significant population differentiation (F_st_=0.45) between Asian and European pears. Interspecific hybrids displayed an average of 55% and 45% introgression from their Asian and European ancestors, respectively. Phenotypic (firmness, acidity, shape and so on) variation between accessions was significantly associated with genetic differentiation. Allele frequencies at large-effect SNP loci were significantly different between genetic groups, suggesting footprints of directional selection. Selection scan analyses identified over 20 outlier SNP loci with substantial statistical support, likely to be subject to directional selection or closely linked to loci under selection.

## INTRODUCTION

Pear belongs to the genus *Pyrus* in the family Rosaceae, and has a basic chromosome number of *x*=17. The number of catalogued species, most of which are diploid (2n=34), in the genus *Pyrus* vary according to different studies, but there could be as many as 75 species.^1^ It is believed that genus *Pyrus* originated during the Tertiary period (65 to 55 million years ago) in the mountainous regions of western China. Evidence suggests that pear dispersion and speciation followed the mountain ranges to both the east and the west.^2^ The ancient Romans made a great contribution to pear domestication by developing methods of propagation, grafting and caring for fruit. There were reported to be more than 40 cultivars existing in the 1st century B.C.^1^ Pear has been cultivated for at least 2000–3000 years, and is currently grown commercially in >50 countries in Europe, Northern Africa, Asia, Australasia and North America.^3^ One of the main reasons breeding programmes are present in almost every continent is because it is important to have cultivars adapted to their growing environment. In spite of the wide geographical distribution of the genus, there are no major incompatibility barriers to interspecific hybridisation. Interspecies hybrids are sometimes developed in pear breeding programmes to produce new cultivars with novel combinations of texture and flavour, and to improve resistance to pests and diseases.^4,5^

Molecular markers have become the preferred tools for characterising genetic diversity. The most frequently used method to assess population differentiation is the calculation of F_st_, a summary statistic that quantifies the variation in marker allele frequencies between populations.^6^ Genetic diversity and genetic relatedness studies within and between species in Asian pears identified markers specific to species, and the clustering of species was largely in agreement with their geographic distribution.^7–9^ Genetic analysis of 145 wild and cultivated accessions of *P. communis* clearly separated accessions native to the Caucasus Mountains from those native to Eastern European countries.^10^ Clustering patterns corresponding with geographic origin were also observed among *P. communis* accessions collected from 12 provenances in Northern Spain.^11^

Studies on genetic diversity among Asiatic and European pears revealed three genetic groups, with the primary division between occidental (Europe and Central Asia) and oriental (East Asia) pears, followed by division of Japanese and Chinese accessions.^7,12,13^ Artificial as well as natural interspecific hybridisation have resulted in complex population structures of pear accessions. Bayesian inference of population structures showed that Japanese *P. ussuriensis* was genetically admixed with two genetic clusters: true native *P. ussuriensis var*. *ussuriensis* and prehistorically introduced *P. pyrifolia*.^14^ Clustering patterns of some *P. communis* accessions from Turkey and Macedonia indicated gene flow and introgression resulting from co-occurring congeneric subspecies.^10^ Some earlier studies using dominant markers revealed that the Chinese sand pear (*P. pyrifolia*) and the white pear (*P.×bretschneideri*) might share a common ancestor.^8^

*Pyrus* diversity studies to date have relied on a limited number of markers (<150). Using a small number of markers can only detect genetic diversity of limited regions of the genome, and could lead to biased or misleading inferences about F_st_.^15^ Moreover, simulation^16,17^ and empirical^18^ studies have shown that SSR loci are likely to produce a significant downward bias in estimates of F_st_ because of the mutational characteristics of highly polymorphic microsatellites. Genome-wide dense genotyping of *Pyrus* species should offer a method of obtaining more reliable estimates of genetic diversity. Wu *et al.*^19^ published the draft genome (512.0 Mb) of the Chinese pear cultivar ‘Dangshansuli’ (*P.×bretschneideri*), which was followed by the publication of a draft genome (577.3 Mb) of the European pear cultivar ‘Bartlett’.^20^ These resources provided opportunities to develop high-density genotyping platforms such as genotyping-by-sequencing (GBS), which is a reduced representation sequencing technology (which involve digestion of genomic DNA with specific restriction enzymes) currently being used for linkage map construction, genomic selection, genome-wide association (GWA) analysis and genetic diversity studies in various plant species.^21,22^

When a large number of genome-wide markers are genotyped across multiple populations, empirical distribution of F_st_ values can be used to identify loci that have been affected by selection.^23^ Such resources can also facilitate the identification of private alleles as well as genomic regions subject to distinct selective environments in geographically separated populations. The New Zealand Institute for Plant & Food Research Limited (PFR) pear breeding programme is diverse, spanning European and Asian species as well as hybrids between these species in an attempt to create new cultivars. The main objective of this study was to conduct a GBS survey of accessions representing European and Asian pear species and interspecific hybrids, to assess genetic diversity, phylogeny and population structure. In this work, we describe the analysis of genome-wide single-nucleotide polymorphic (SNP) allele frequency differences between populations, which provides a powerful approach to interrogate the genome for signatures of selection. We also used GWA analysis to identify genomic regions underlying genetic variation in several fruit traits.

## MATERIAL AND METHODS

### Plant material and phenotypes

Accessions of various pear species were imported from around the world and crossing between European and Asian species and among Asian species commenced in 1983.^24^ In addition to the imported accessions, a large number of advanced selections made from the hybrid families are also propagated on to Quince C or Quince BA29 rootstock, interstocked with ‘Beurre Hardy’, in PFR’s Pear Repository. For the purpose of this study a total of 214 accessions, including 35 *P. pyrifolia*, 9 *P.×bretschneideri*, 1 *P. pashia*, 1 *P. betulaefolia*, 2 *P. calleryana*, 112 *P*. *communis,* and 54 interspecific hybrids (Supplementary Table S1) were sampled. Young leaves were collected in spring 2013 for DNA extraction.

Fruit were harvested in the fruiting season (February to May) in 2014 and 2015 when fruit background colour was beginning to change from green to yellow. Six fruit from each seedling were stored for 28 days at 3 °C, then a further 1 day at 20 °C before evaluation. Phenotypic information on traits describing visual, sensory and instrumental fruit properties was obtained, and the six fruit were given one overall score for each trait. Briefly, skin russet coverage (RUS) and skin bitterness (BIT) were scored on scales 0 (none) to 9 (highest). Skin over-colour coverage was analysed as a presence (red)/absence (no red) trait. Scuffing (SCUF) was rated on a 0–9 scale (0=no darkening; 9=solid brown or black colouration) after each fruit was firmly rubbed across the cup of a moulded pulp fibreboard fruit packing tray and assessed after 2 h.^25^ Fruit shape index was visually scored using a shape chart developed in-house (Supplementary Figure S1). Fruit firmness (FF) was determined on opposite sides of each fruit after peel removal using a Fruit Texture Analyzer (GÜSS) fitted with an 11-mm diameter probe tip. Bulked juice from the cortical flesh of the sample fruit was used to measure titratable acidity (TA) using an automatic acid titrator (Metrohm 716 DMS).

### Phenotypic data analysis

Estimates of variance components for each trait were obtained using the following linear mixed model:

$$y=Xb+Z_{1}a+Z_{2}t+e$$

where ***y*** is a vector of phenotypes on a trait; ***b*** is a vector of fixed effects (that is, the intercept, year); $a∼N(0,\;G\sigma_{a}^{2})$ is a vector of random additive effects of accessions with variance $\sigma_{a}^{2}$; ***G*** is the additive relationship matrix; $t∼N(0,\;I_{s}⊗G\sigma_{\mathrm{as}}^{2})$ is a vector of random interactions of accessions (*a*) with year (*s*); $\sigma_{\mathrm{as}}^{2}$ represents interaction variance; ***I***_s_ represents an identity matrix with order equal to the number of years; ⊗ denotes the Kronecker product operation; *X*, *Z*_1_ and *Z*_2_ are incidence matrices for the fixed effects, random accession effects, and interaction effects, respectively; $e∼N(0,\;\sigma_{e}^{2})$ is a vector of random residual terms with variance $\sigma_{e}^{2}$. The ***G*** matrix was constructed using SNP marker information according to method from a previous study.^26^ ASReml software ^27^ was used for estimation of variance components. Ratio of the additive variance $\left(\sigma_{a}^{2}\right)$ to the phenotypic variance $\left(=\sigma_{a}^{2}+\sigma_{\mathrm{as}}^{2}+\sigma_{e}^{2}\right)$ was interpreted as heritability (*h*^2^).

### DNA extraction and GBS library preparation

Total DNA (DNA) was extracted from leaf material after ball bearing milling (Omni Bead Rupter, Omni International), for 1 min at 3.55 m s^−1^ in a CTAB based buffer.^28^ The homogenate was incubated at 65 °C for 30 min, cooled and a chloroform extraction performed. The samples were centrifuged to separate the chloroform phase and insoluble plant material from the aqueous phase layer containing the DNA. This aqueous phase was pipetted into a new tube and the DNA was precipitated with the addition of 2/3 vol. isopropanol and centrifuged for 10 min at 14000 *g*. The DNA pellet was washed two times with 70% ethanol, lightly dried and finally resuspended in TE (10/0.1) buffer. The DNA concentrations were determined by fluorimetry (Qubit, Life Technologies, Waltham, MA, USA) according to manufacturer's instructions. One hundred nanograms of each DNA sample was electrophoresed (3–4 V cm^−1^) on a 1% (w/v) agarose gel in TAE buffer, with in-gel staining using RedSafe (iNtRON Biotechnology, Korea) and UV illumination for visualisation to determine the quality level of intact, high molecular weight DNA.

GBS libraries were prepared for each DNA sample using a small modification on the protocol developed by Elshire *et al.*^21^. Ninety-six bar-code adaptors designed by Deena Bioinformatics,^29^ the + and − strand oligonucleotides, set out in plate format were annealed, quantified by high-sensitivity double-stranded-DNA-specific fluorimetry (Qubit) and the concentrations were normalised. A common adaptor, + and − oligonucleotides, were also annealed and quantified, and added to each bar-code adaptor well. The final working concentrations of each adapter per well was 0.3 ng/ μl. Six microlitres of this adaptor mix was pipetted to a new 96-well plate and dried down under light vacuum (CentriVap concentrator, Labconco, Kansas City, MO, USA). One hundred nanograms of DNA from a plant sample was aliquoted into a well and also dried down. The samples were re-suspended in a digest cocktail of *BamHI* type II restriction endonuclease (New England Biolabs, Ipswich, MA, USA) and incubated according to manufacturer’s instructions. The barcoded and common adaptors were ligated to the digested DNA with a T4 DNA ligase (Promega, Madison, WI, USA) cocktail and incubated according to the manufacturer’s instructions. The ligation of the adaptors to the cut ends of the DNA does not re-create the restriction enzyme site. Then 2.5 μL of each library was amplified following Elshire *et al.*^21^ with primers (PPA and PPB) that annealed to sites on the adaptors using a high fidelity DNA polymerase (Life Technologies). After an initial denaturation step of 95 °C, 2 min, reactions were then cycled (95 °C, 30 s→65 °C, 30 s→68 °C, 30 s) 25 times before a final elongation step of 68 °C, 5 min. An aliquot of each amplification was electrophoresed on a 3% agarose gel and stained with ethidium bromide before visualisation under UV illumination to examine the library fragments. The remaining volume from each library amplification were pooled without normalisation and cleaned up on a PCR clean-up column (Qiagen, Hilden, Germany) prior to sequencing. GBS libraries were multiplexed into 5 pools, with 36 to 55 libraries per pool, for next-generation sequencing (NGS).

### Variants discovery from GBS data

The sequencing was performed at Macrogen Inc., Seoul, Republic of Korea. Each pool of GBS libraries was sequenced in one lane, on the Illumina HiSeq2000 platform (San Diego, CA, USA), in single-end mode. The read length was 101 bases and the output was 165 to 203 million reads per lane. The quality of the original sequencing files was examined using FastQC (version 0.11.1; Babraham Bioinformatics; Cambridge, UK) to ensure that the data yield was acceptable and the quality was satisfactory with Phred scores >20 along the reads. A TASSEL^30^ compatible key file was constructed for all plates based on the plate layout of the GBS library preparation, the sequencing flowcell code and the sequencing lane for the plate using PERL script that was developed in-house. Five lanes of GBS data were analysed simultaneously using TASSEL/GBS pipeline on the Linux platform to discover SNP falling within 64 bases of a *BamHI* site. The sequencing reads were converted to TASSEL tags using the *FastqToTagCount* plug-in that requests at least three supporting reads for a tag. Duplicate tags from different lanes were merged with the *MergeMultipleTagCount* plug-in. Along with the raw sequencing data and the key file, the tags were separated according to samples using *SeqToTBTHDF5* plug-in to generate tag-by-taxa (TBT) results. The merged unique tags were converted to Fastq format through *TagCountToFastq* plug-in.

After the tag Fastq file was constructed, two analyses were carried out, with *P.*×*bretschneideri* (cultivar ‘Suli’) and *P. communis* (cultivar ‘Bartlett’) as reference genomes, respectively. In each analysis, the Fastq file was mapped to the reference genome using Bowtie2 (version 2.2.1)^31^ in the ‘very-sensitive-local’ mode. The alignments were converted into the tag-on-physical-map (TOPM) format using the *SAMConverter* plug-in, which was followed by the *ModifyTBTHDF5* plug-in for efficient SNP calling. On the basis of the TBT and TOPM results, SNP sites were called using the *TagsToSNPByAlignment* plug-in with ‘minimum minor allele frequency’ set to 0.001, ‘minimum minor allele count’ to 3, ‘minimum locus coverage’ to 0.1 and allowing rare alleles calls at site. To improve the reliability of SNP calling, filtering was applied using the *GBSHapMapFilters* plug-in with ‘minimum site coverage’ set to 0.9.

The publically available genetic map of an interspecific family,^32^ and a new GBS-based map of a *P. communis* family,^33^ were then used to assign scaffolds to different linkage groups (LG). Details describing the genetic maps were retrieved from these two papers, and converted to tab-delimited text format with columns: Marker name; Scaffold ID; Map position (cM on LG), LG; and Physical position (on scaffold). For each scaffold in the genetic maps, the number of SNP and LG identifiers were summarised using PERL script. If a scaffold had all its SNP markers mapped onto one LG in the genetic maps, the scaffold was assigned to that LG and classified as type I scaffold. If SNP on one scaffold were assigned to multiple LGs, the scaffold was classified as type II scaffold. All SNP detected on *P. communis* type I scaffolds (based on the Li *et al.*^33^ genetic map) were retained. Similarly, SNP on *P.×bretscheneideri* type I scaffolds in the Wu *et al.*^32^ genetic map were kept for further processing. Multi-allelic SNP were discarded, along with SNP with minor allele frequency (MAF)<0.025, and missing data frequency >10%. Missing genotypes at the remaining SNP loci were imputed using LinkImpute software,^34^ which implements a *k*-nearest neighbour genotype imputation method designed for unordered markers.

### Population structure and linkage disequilibrium analysis

We first constructed an un-rooted neighbour-joining (NJ) tree, which is an empirical description of a distance matrix, using the R package ‘ape’^35^ with default settings. The population structure was investigated using the model-based Bayesian clustering method implemented in STRUCTURE,^36^ which uses Markov Chain Monte Carlo simulations to infer the proportion of ancestry of genotypes in *K* distinct predefined clusters. Ten independent runs were carried out for different *K* parameter values (*K*=1 to 4), and we used 50 000 Markov Chain Monte Carlo iterations after a burn-in of 5000 steps. Principal component analysis of the genotypic data matrix was also conducted to evaluate clustering patterns of all 214 accessions. Pairwise linkage disequilibrium (LD) between SNP markers was calculated to evaluate the extent of LD decay. The degree of LD was quantified with the parameter *r*^2^ obtained by taking into account the population structure and cryptic relatedness using R software ‘LDcorSV’ version 1.3.1.^37^

### Evidence of selection

Population genetic parameters including observed heterozygosity (*H*_o_), gene diversity (*H*_s_) and a measure of allele frequency differences between genetic groups (F_st_) were calculated at each SNP locus, and also across all loci, using R package ‘hierfstat’.^38^ Genome scans for outlier F_st_ values, as an evidence of selection, were also conducted using BayeScan software.^39^ For this purpose, F_st_ coefficients are decomposed into a population-specific component (beta), shared by all loci and a locus-specific component (alpha) shared by all the populations using a logistic regression. A positive value of alpha suggests diversifying selection, whereas negative values suggest balancing or purifying selection. BayeScan estimates the probability that a locus is under selection by calculating a Bayes factor (BF), which is the ratio of the posterior probabilities of two models (selection/neutral) given the data. A BF between 3 and 10 (Log_10_(BF)=0.5–1.0) is considered as a ‘substantial evidence’ of different statistical support for the two models.^39^

We also tested for genome-wide signals of marker-phenotype association to determine whether the loci of functional (associated with economically important traits) significance coincided with the outlier loci. We used the best linear unbiased estimate (BLUE), adjusted for year effect, as the phenotypic traits.^40^ BLUE were calculated by fitting the following fixed-effects model in ASReml software:^27^ [phenotype=mean+year+accession+(year×accession)+residuals]. Marker-trait association analyses were conducted using a mixed linear model approach implemented in GAPIT.^41^ A realised relationship matrix (K-matrix) and covariates from Q-matrix (derived from principal component analysis), calculated by GAPIT, were used as a correction for cryptic relatedness and population stratification, respectively, in the association models.

## RESULTS

### Estimates of variance components and heritability

The additive variance $\left(\sigma_{a}^{2}\right)$ was the major source of variability for all traits except TA and BIT (Table 1). On average, the additive variance accounted for 55% of the phenotypic variation. For various traits, the magnitude of genotype-by-year interaction variance varied between 0 (SCU, COL) and 29% (Shape), with an average of 14% (Table 1). The heritability estimate (*h*^2^) was low (<0.20) for TA, moderate (0.20–0.40) for BIT, and high (>0.40) for all other traits (Table 1). The skin over-colour was the most highly heritable trait (*h*^2^=0.86).

### GBS and SNP calling

Using ‘site coverage’ of 0.1, a total of 597 032 and 395 710 SNP markers were detected on *P. communis* and *P.×bretschneideri* genomes, respectively. Using a higher site coverage (0.9) drastically reduced the number of detected markers to 54 121 on 1979 *P. communis* scaffolds and to 47 821 markers on 776 *P.×bretschneideri* scaffolds. The next step was to assign these 2755 (=1979+776) scaffolds to LGs according to the ‘Suli’^32^ and ‘Bartlett’^33^ maps. If a scaffold had all markers mapped onto one LG in the corresponding map, the scaffold was assigned to that particular LG (and classified as type I scaffold). Following this approach, 597 type I scaffolds (out of 2755) were assigned to a particular LG. Scaffolds on the same LG were ordered by their map position (cM) in the genetic maps. SNP loci detected on these type I scaffolds were kept for further quality checks.

**Figure 1:**
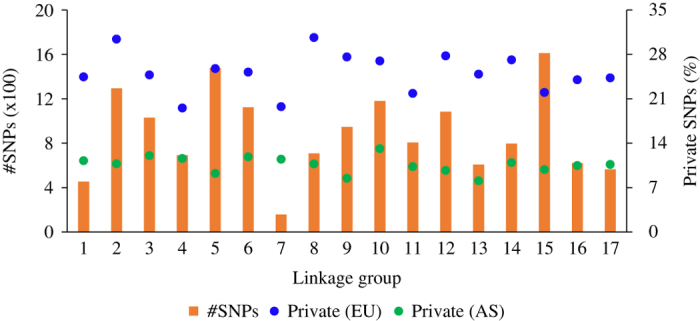
Distribution of retained SNP markers across different linkage groups. The frequency (%) of SNP markers private to the European (EU) and Asian (AS) species is shown, for each linkage group (*x *axis), on the secondary *y *axis.

The median read depth per SNP locus was 118, and SNP loci with read depth <8 or >1000 were removed. After some additional filtering criteria (number of alleles, MAF and missing data frequency), the number of retained SNP markers varied from 157 (on LG7) to 1,610 (on LG15), with a total of 15 146 (Figure 1). About 9% of these SNP were common to those mapped previously to the linkage maps of *P.×bretschneideri* and *P. communis*. Across all 214 accessions, the MAF at various SNP loci varied between 0.025 and 0.50, with an average of 0.18. However, about 25 and 11% of all markers were found fixed in the European (*P. communis*) and Asian (all species other than *P. communis*) pear groups, respectively, while all SNP loci were heterozygous in the hybrid group. The number of fixed loci was fairly consistent between LGs, that is, ranging from 20% to 30% and 8% to 13% for the European and Asian species, respectively. The difference between the Asian and European pears, in terms of the number of private alleles, was largest (139 vs. 393, respectively) on LG2 and smallest (18 vs. 31, respectively) on LG7 (Figure 1). Different allele frequencies in different genetic groups can result in most loci out of Hardy–Weinberg equilibrium. Thus, SNP markers departing from H-W equilibrium were not discarded, however, population structure was taken into account for further analyses.

### Population differentiation, structure and LD

**Figure 2:**
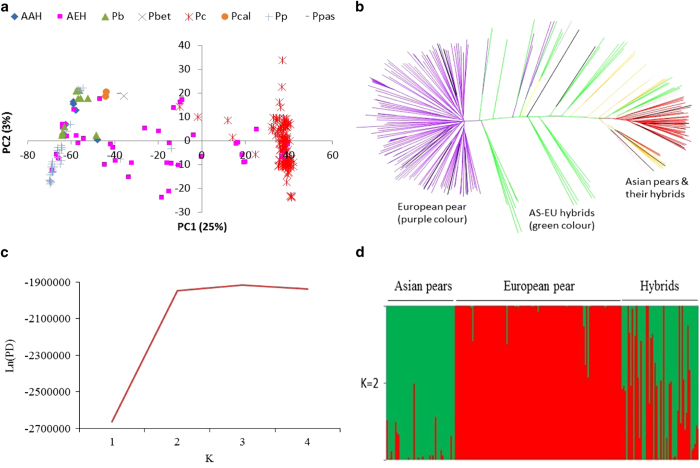
Population structure analysis using principal component (**a**), neighbour joining (**b**) and Bayesian clustering (**c**, **d**). AAH: Asian-Asian hybrid; AEH: Asian–European hybrid; Pb: *P. ×bretschneideri*; Pbet: *P. betulaefolia*; Pc: *P. communis*; Pcal: *P. calleryana*; Pp: *P. pyrifolia*; Ppas: *P. pashia*. (**c**): The likelihood of the posterior density (Ln(PD) is shown for various numbers of clusters (*K*). (**d**): The mean estimated membership probability (*y* axis) of each accession (*x* axis) to the two clusters (*K*=2).

The first principal component (PC1) grouped the accessions belonging to Asian and European species in two non-overlapping clusters. Hybrids among the Asian species clustered within their parental group, but the hybrids between Asian and European pears resided in between the two main clusters depending on the degree of introgression from the parental species (Figure 2a). PC2 revealed the variability within each of the three genetic groups (Asian, European and Hybrid). A break-away group of a few accessions of *P.×bretschneideri* and *P. pyrifolia* formed a sub-group within the Asian cluster (top left-hand corner of Figure 2a) where representatives of some of the other Asian species also co-located. The genome-wide gene diversity (*H*_*s*_) was 0.25 for the Hybrid group, which is slightly higher than the European (0.21) and Asian (0.20) pears. The overall observed heterozygosity (*H*_*o*_) was very similar for the Asian (0.18), European (0.20) and hybrid (0.18) genetic group.

The genome-level population-differentiation statistic (F_st_) between Asian and European pears was estimated at 0.44, indicating a very strong population differentiation. The overall estimated F_st_ between the European and Hybrid groups was 0.21, which is twice that between the Asian and Hybrid pears (0.10). The distribution of F_st_ values between pairs of genetic groups is shown in Supplementary Figure S2. F_IS_, which measures inbreeding, was highest in the Hybrid group (0.28) followed by the Asian (0.18) and European pears (0.05). High F_IS_ values at the group level also indicate a cryptic population structure within each group, which is also supported by the branching patterns of accessions within each major group on a phylogenetic tree (Figure 2b). The NJ tree reflected larger genetic distances between the European and Asian accessions, whereas hybrids were generally in the middle of the two genetic groups (Figure 2b).

**Figure 3:**
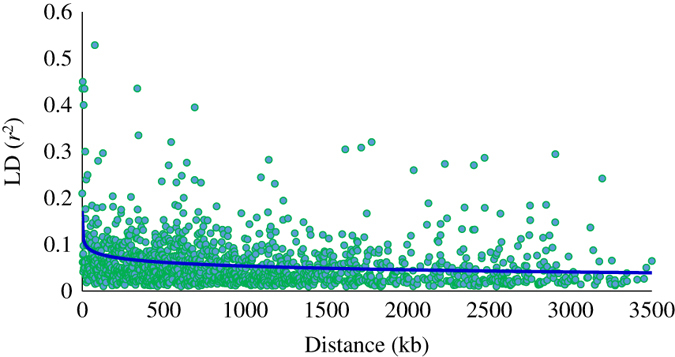
Average (across all scaffolds) linkage disequilibrium (LD; *r*^2^) values. Decay of LD with distance (kilobase) was estimated from a logarithmic trend line (blue colour).

Admixture analyses were run for population structure models assuming *K* (the number of clusters)=1 to 4. The likelihood of the posterior density (Ln (PD) value changed very little for *K*>2 (Figure 2c) and the Δ*K* statistic, designed to identify the most relevant number of clusters, was the highest for *K*=2. The mean estimated membership of the Asian accessions to the two clusters, namely Asian pears and European pear, was 96% and 4%, respectively (Figure 2d); whereas the membership of the European accessions to these clusters was 1% and 99% respectively. The interspecific hybrids displayed an average 55 and 45% introgression from the Asian and European species, respectively (Figure 2d). All available accessions (214) were used for calculating the LD statistics (*r*^2^) between pairs of markers on the same scaffold. The average within-scaffolds LD (*r*^2^) values were 0.20, 0.10, 0.07 and 0.05 for markers separated by 10, 100, 500 and 1000 kb distance, respectively (Figure 3).

### Evidence of selection and genotype–phenotype association

**Figure 4:**
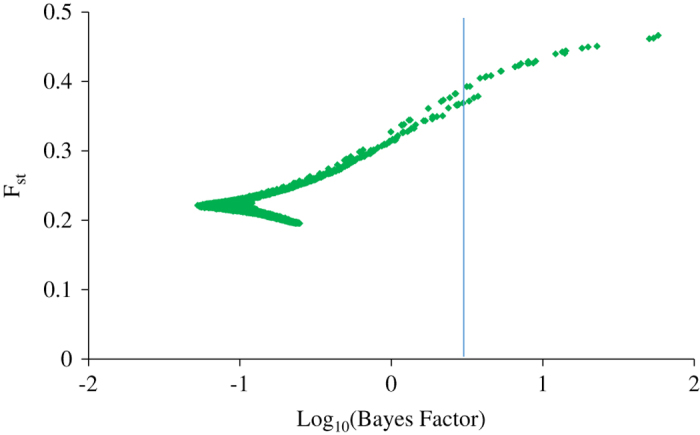
Genome-wide scans for identification of F_st_ outlier loci potentially subject to differential selection. Shown are Log transformed Bayes factors (BF) and locus-specific F_st_ from BayeScan. Vertical line mark Log_10_(BF) of 0.5 corresponding to posterior probability of locus effects of 0.95.

A total of 24 SNP loci located on LGs 2, 3, 6, 10, 12, 14, 15 and 16 displayed substantial selection signatures (log10 Bayes Factor >0.5), consistent with a model of directional selection at the associated SNP marker or other closely linked genes (Figure 4; Table 2). None of the significant loci displayed balancing or purifying selection. A separate analysis of only the Asian and European groups did not identify any new SNP in addition to those identified from analysis of all three genetic groups together, suggesting that selection signatures in our study are dominated by allele frequency differences between parental species and their hybrids.

**Figure 5:**
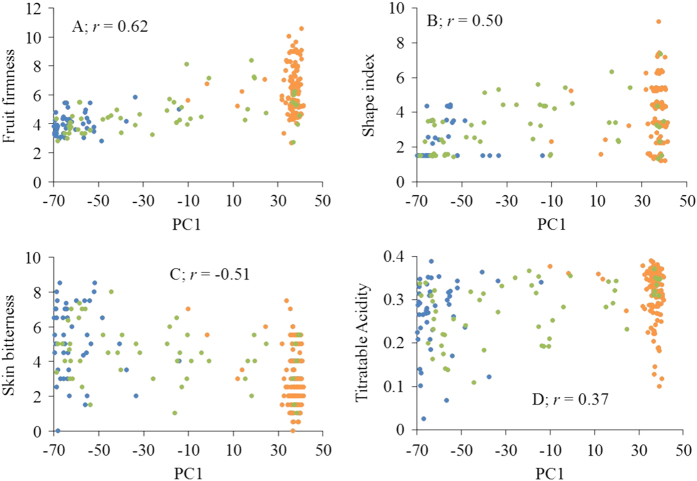
Genotype–phenotype correlation (*r*). *X *axis: first principal component (PC1) of genome-wide marker scores of accessions; *Y* axis: phenotypes of Asian (blue), Hybrids (green) and European (orange) accessions.

For some fruit quality traits, phenotypic variation was significantly correlated with genetic differentiation (that is, PC1 scores) between the three groups. The highest correlation (0.62) was observed between fruit firmness and PC1 scores (Figure 5). GWA showed that the majority of individual markers explained only a small proportion of phenotypic variation (≈0.5%), while the largest-effect SNP explained 8%, 8%, 6%, 10%, 9% and 6% of the phenotypic variation for firmness, TA, shape, russet, scuffing and skin over-colour, respectively (Table 3 and Supplementary Figure S3). Our study also found markers, located on LGs 3, 5, 9 and 17, each accounting for about 5% of variation in fruit firmness. The three markers most strongly associated with the presence of red skin colour were located on LGs 9, 14 and 5, each explaining about 4–6% of the phenotypic variation (Supplementary Figure S3 and Table 3). Quantile–quantile (QQ) plots, comparing GWA probability with that expected under the null hypothesis of no association, are shown for each trait in Supplementary Figure S4. There was no highly significant SNP associated with skin bitterness.

The frequency of alleles at the largest-effect SNP loci displayed large differences among the three genetic groups (Table 3). For example, the alleles associated with firmness and over-colour at loci S71.0_485233 and S155.0_757109, respectively, were only present in European accessions. Similarly, the alleles associated with TA, russet, and scuffing at loci S895.0_79244, S398.0_140201 and S149.0_728797, respectively, were only present in Asian accessions. The allele associated with fruit shape was present in both European and Asian accessions. The flanking DNA sequences of the largest-effect SNP loci are provided in Supplementary Figure S5.

## DISCUSSION

### Species differentiation and genetic diversity

GBS data offers the promise of revealing complex demographic scenarios, and an assessment of gene flow and introgression effects on genetic diversity. The GBS approach implemented in our study revealed a large number of alleles that were private to either of the two major species groups (European and Asian), which further suggested the suitability of GBS for biodiversity conservation of *Pyrus* spp. and identification of fine-scale introgressions in interspecific hybrids. Private alleles of some Asian pear species were reported earlier using limited numbers of SSR markers,^42^ but our study provides a much better overview of genome-wide regions harbouring private alleles of European and Asian pears. Geographic isolation of European and Asian pears would suggest independent evolution of these species and thus it is not surprising to find a large number of private alleles in the respective populations. The reproductive isolation of populations is thought to be an initial step towards speciation.^43^ Our findings of adaptation and/or speciation-associated SNP markers are only preliminary, which upon further validation could help in the better management and conservation of genetic resources of European and Asian pears.

The admixed nature of the hybrid group was reflected in its relatively higher gene diversity (*H*_*s*_) than the ancestral species. The genome-wide inbreeding coefficient (F_IS_) of the hybrid group was the highest (0.28), followed by the Asian (0.18) and European (0.05) group of accessions. These results suggested that each genetic group deviated from panmixia, which is consistent with a common practice of assortative mating in pear breeding programmes. F_IS_ values were reported to vary considerably (0.06–0.18) in wild populations of *P. ussuriensis*.^44^ On the basis of 135 SSR markers, Liu *et al.*^7^ reported an average F_IS_ value of 0.23 across accessions of Asian and European pears. These results indicate somewhat higher F_IS_ coefficients of cultivated/advanced accessions compared with wild populations.

Genetic differentiation (F_st_) between hybrids and European pear was twice of that between hybrids and Asian pears (0.21 vs 0.10). A key focus of our interspecific breeding programme is to introgress fruit quality traits (for example, crisp textures, resistance to scuffing, spur bearing, long shelf life) from the Asian gene pool into hybrid cultivars, which could partly explain relatively lower F_st_ between these two groups. Introgression sites are indicated where F_st_ values approach zero, that is, where there is no divergence between the regions of hybrid and their ancestral species.^45^ The number of introgression events from the Asian gene pool were higher than the European gene pool, and these events were evident on most LGs (Supplementary Figure S2). The percentage of admixed ancestry of the hybrid group in the Asian gene pool was 55% (compared with 45% in the European group), which supports relatively lower genome-wide F_st_ between these two genetic groups. Understanding the precise position and size of introgressions will help develop molecular markers to reduce linkage drag from donor species.^46,47^

Divergence between the European and Asian species illustrated substantial variation (F_st_ ranging between 0 and 0.99) across the whole genome, which is supported by their geographic isolation. F_st_ values close to 1 suggest reproductive isolation and a high inter-group divergence relative to intra-group diversity.^45,48^ Genome-wide overall estimated F_st_ between the Asian and European pears was 0.44, which is much higher than earlier (cf. 0.15) reports.^7,49^ Being based on only a small number (<150) of markers, earlier studies could have underestimated the genome-wide differentiation. Geographic isolation, local adaptation and population-specific directional selection could have accentuated population differentiation, thus resulting in high observed F_st_ in our study. Allele frequency differences between parental species and artificial hybrids (Table 3) would amount to artificial selection.

### Population structure

Bayesian model-based clustering was conducted to estimate the population structure of 214 *Pyrus* accessions. The most relevant number of clusters was found to be 2 (Asian and European). The membership coefficient of the European and Asian pears to these two main clusters (0.99 and 0.96, respectively) suggested a minimal gene flow between the two groups—probably because of their geographic isolation. This low level of gene flow is also a primary cause of high genetic differentiation F_st_. The average membership coefficient of hybrids to the Asian and European clusters was 0.55 and 0.45, respectively, but a substantial variation in their membership coefficients was also observed. A small number (<10) of first-generation European-Asian hybrids displayed a high membership coefficient (close to 1.0) of either the Asian or European cluster, suggesting extreme cases of apparent segregation distortion. Distorted segregation, generally observed in interspecific hybrids, could be because of high divergence between parental genome sequences—leading to DNA mismatches during meiotic recombination, which can in turn disrupt meiotic crossovers and accurate chromosome segregation.^47,50^

Some *P. communis* cultivars, namely ‘Tosca’, ‘Tenn’, ‘Moders’, ‘Florida Home’ and ‘Jupp’ displayed introgression from Asian pears (Figure 2d), while a *P.×bretschneideri* accession (‘Qiyuesu’) and a *P. pyrifolia* accession (‘Hokusei’) displayed membership coefficients of 0.15 and 0.27, respectively, of the European cluster. One accession (P02) supposedly derived from a cross between *P. pyrifolia* cultivars (‘Nijisseiki’×‘Kosui’) was placed half-way between the Asian and European clusters (Figures 2a and d). These results suggested some discrepancy with their documented ancestry. In our study, we had two open-pollinated (OP) seedlings representing *P. calleryana*, and one seedling each of *P. pashia* and *P. betulaefolia*. These four accessions displayed moderate degree of membership coefficient (0.17–0.25) of the European cluster, suggesting that these seedlings could have originated from cross fertilisation with *P. communis*.

First principal component (PC1) cleanly separated *P. communis* from Asian pears, which was further supported by a large number of fixed (or private) alleles for these two gene pools (Figure 2a and Figure 1). There were patterns of subgrouping within the Asian pears cluster. For example, five of the *P.×bretschneideri* accessions (‘Xinyali’, ‘Yali’, ‘Tsuli’, ‘Xuehuali’ and ‘Pingguoli’) grouped separately with three red skin accessions (‘Winshan’ OP, ‘Huobali’ OP, and ‘Yanshan’ OP) of *P. pyrifolia* ancestry. ‘Pingguoli’ is a red skin cultivar and similar to some other cultivars (for example, ‘Yali’ and ‘Tsuli’), has also been classified as *P.×bretschneideri* or *P. pyrifolia* in different studies.^7,51,52^ The genomes of *P. ussuriensis* and *P. pyrifolia* are thought to have contributed to the origin of *P.×bretschneideri*, so it is not surprising to observe overlapping clustering of accessions of these species.^8^

‘Beurré Hardy’, which is commonly used as interstock between pear scions and quince rootstocks, was located (top right-hand quadrant Figure 2a) away from the rest of the European pear accessions. A sub-group of *P. communis* accessions was also noticeable (lower right-hand quadrant Figure 2a), which consisted of ‘Bartlett’ bud mutants, namely ‘Max Red Bartlett’, ‘Red Sensation Bartlett, ‘Swiss Bartlett’ and ‘Jumbo Starks’. These results suggested that the genome-wide DNA profiles (that is, genetic constituent) of these ‘Bartlett’ sports are near identical despite some mutants having red-skin or very large fruit size. Wang *et al.*^53^ showed that the coding regions of *PcMYB10* were the same between red and green sports of ‘Max Red Bartlett’, but methylation of the promoter region of *PcMYB10* repressed the expression of *PcMYB10* and subsequently inhibited the biosynthesis of anthocyanin, which probably caused the formation of a green-skin sport of ‘Max Red Bartlett’. Qian *et al.*^54^ also made similar observations for red and green-skin sports of the pear cultivar ‘Zaosu’, suggesting that inter-retrotransposon amplified polymorphic (IRAP) markers would be better suited to differentiate between bud sports and their original cultivars.^55^

### Trait architecture

Fruit scuffing was among the highly heritable (0.61) traits in this study. Brewer *et al.*^25^ also reported a high heritability (*h*^2^=0.72) for scuffing and Saeed *et al.*^56^ mapped numerous QTLs for scuffing, including those on LGs 2 and 15, which supports results of this study and highlights the complex polygenic architecture of this trait. We identified a large-effect SNP (on LG8) associated with fruit russet skin. In previous studies, a marker associated with fruit russet skin was mapped to LG8.^57,58^ Similarly, Song *et al.*^59^ showed that the pear fruit russet skin trait is linked to apple SSRs CH01c06 and Hi20b03 located on apple LG8.

Fruit firmness, which is often a key selection trait in pear cultivar breeding programmes, is a highly heritable trait,^60^ but QTL mapping studies^56,58^ did not identify any large effect QTL for this trait. GWA analysis in this study identified a new QTL for fruit firmness on LG16, and the next best SNP explaining about 5% of variation in fruit firmness was located on LG15, where two ethylene producing genes (*PpACS1 and PpACS2*) were identified in an earlier study.^61^ The most significant SNP, explaining 8% of variation in TA, was located on LG2, which is the same one as that of an earlier study using a bi-parental cross between European and Asian species.^62^ The next best SNP associated with TA was located on LG7 (Supplementary Figure S3), which appears to be a new genomic region that has not been reported before. Most genomic regions found significantly associated with fruit shape index were unique, but a QTL on LG2 could coincide with an earlier reported QTL in a bi-parental family.^63^

Functionality of *PcMYB10*, which can activate the expression of genes encoding enzymes of the anthocyanin biosynthetic pathway leading to red skin colouration of pear fruit, was verified by transient expression assay.^64^ In a cross between ‘Abbé Fétel’ and ‘Max Red Bartlett’, Pierantoni *et al.*^65^ mapped *PcMYB10* on LG9 of both cultivars. Our analysis identified a SNP associated with the red-skin phenotype on LG9, but it is not clear if this SNP resides in close proximity of *PcMYB10*. Although *PcMYB10* appears to play a role in the pigmentation of the pear fruit skin, other *MYB* genes in combination with sunlight and temperature could also contribute to the phenotypic variation in pear red skin colour variation. For example, Dondini *et al.*^66^ mapped a major gene associated with red skin colour on LG4 of the ‘Max Red Bartlett’ map. QTL for red skin colour have also been mapped on LG16,^32^ suggesting a complex polygenic nature of this trait.

GWA is a powerful technique for detecting functional variants based on the association between genome-wide markers and phenotypes caused by LD between markers and causal genes or QTL. The maximum phenotypic variance explained by individual SNP markers was less than 10% in this study, probably due to faster LD decay in wider germplasm compared with family-based designs.^67^ The extent of LD in a sample of Japanese cultivars, reported by Iwata *et al*.,^68^ was almost twice that observed in our study, which could mainly be due to the large diversity and admixture nature of our study population. Due to faster LD decay, GWA in admixed populations is far more challenging than in homogeneous populations and requires a relatively higher marker density and population size.^69,70^

### Selection footprint

Genome scans and F_st_ outlier approaches can be effective at identifying genes under selection without known phenotypes. Extreme differentiation in allele frequencies between genetic groups or geographic zones as measured by the F_st_ provide signatures of recent positive selection.^23^ Relevance of admixed populations (for example, interspecific hybrids) along with ancestral breeds/species have been shown as an attractive biological model to study adaptive or directional selection.^71^ Using an admixed population in this study, we identified loci with log_10_ (Bayes factor) >0.5 (corresponding to ‘substantial’ evidence for selection on the Jeffrey’s scale of evidence for Bayes factors) to show evidence of selection between the three genetic groups (European, Asian and Hybrids) of accessions.

Association between genotypes and phenotypes in differentiated populations provides an additional tool for identifying genomic regions that may form the genetic basis for the observed phenotypic diversity.^72^ Significant correlation between PC1 (which cleanly separated three genetic groups; Figure 2a) and fruit phenotypes (Figure 5) supported the hypothesis that population-restricted artificial selection could have played a role in the observed signature footprints in our study. The fact that alleles influencing certain fruit phenotypes were present in hybrid individuals and absent in one of the ancestral species, suggesting that artificial selection for fruit phenotypes could have played a role in the observed allele frequency differences at these large-effect SNP loci (Table 3). These results provide molecular evidence that a strong directional selection for fruit quality traits in the interspecific breeding programme^4^ have indeed yielded desired outcomes. Brewer *et al.*^25^ observed that Asian accessions were the source of tolerance to scuffing in an interspecific hybrid population. As the allele associated with scuffing at the largest-effect SNP locus was not present in the European accessions (Table 3), our results suggests selection for tolerance to scuffing in Asian pears.

The selection outlier loci in our study did not co-localise with GWA signals. There are various possibilities for these observations. First, it is likely that outlier loci harbour polymorphisms associated with adaptive traits (for example, phenology, reproduction and tolerance to biotic and abiotic stress tolerance) not measured in this study. For example, LG2 harbours large effect QTL for pear fire-blight^73,74^ and polygenic scab resistance.^75^ Second, chromosome-level sequences for the genome of Asian pear (*P.×bretschneideri*) and European pear (*P. communis*) are not yet available, so the genomic coordinates of selection loci and GWA SNP markers are not well defined. Hence, the large-effect SNP identified on LGs 2, 10 and 16 could well be in close proximity of markers from selection scans on these LGs. The number of SNP markers used in this study was sufficient to accurately cluster accessions of different genetic groups. However, an increased SNP density could have helped improve the resolution of differentially selected loci, and improve the concordance between the SNP loci identified from GWA and selection footprint analyses.^72^

## CONCLUSIONS

Selection scans and genotype–phenotype association patterns provided preliminary indication of adaptation and selection footprints. Admixture proportions and genome-wide introgression patterns suggested some extreme cases of segregation distortion in interspecific hybrids derived from crosses between the European and Asian species. As the largest-effect markers only explained <10% of phenotypic variation, the genetic architecture of pear fruit phenotypes appears to be complex and polygenic. Findings from our study have important implications for our understanding of independent evolution of Asian and European pears, and for the conservation of allelic diversity under rapidly increasing pressure from changing climatic and economic conditions.

## Figures and Tables

**Table 1:** Estimates of additive genetic variance $\left(\sigma_{a}^{2}\right)$, genotype-by-year interaction variance $\left(\sigma_{\mathrm{as}}^{2}\right)$, and residual variance $\left(\sigma_{e}^{2}\right)$, expressed as the percentage of phenotypic variance (defined as the sum of variance components in the model)

*Trait*	$\sigma_{a}^{2}$	$\sigma_{\mathrm{as}}^{2}$	$\sigma_{e}^{2}$	h^*2*^
FF	0.67	0.19	0.14	0.67
TA	0.17	0.19	0.64	0.17
Shape	0.58	0.29	0.13	0.58
RUS	0.54	0.27	0.19	0.54
SCU	0.61	0.00	0.39	0.61
OCOL	0.86	0.00	0.14	0.86
BIT	0.39	0.06	0.54	0.39

Abbreviations: FF, fruit firmness; TA, titratable acidity; RUS, russet; SCU, scuffing; OCOL, skin over-colour; BIT, bitterness.

Estimate of heritability (*h*^2^) is also shown for various traits (FF and TA; shape—RUS, SCU, OCOL and BIT).

**Table 2:** Single-nucleotide polymorphic (SNP) markers displaying substantial signature of selection (log10 Bayes Factor>0.5)

*SNP*	*Scaffold*	*Position (basepair)*	*Linkage group*	*Reference genome*	*Gene identity (annotation)*
X2_snp1794	4	361402	2	*P. ×bretschneideri*	Unknown
X2_snp1979	4	1426690	2	*P. ×bretschneideri*	Unknown
X2_snp1992	4	1426910	2	*P. ×bretschneideri*	Unknown
X2_snp906	183	233831	2	*P. ×bretschneideri*	Unknown
X2_snp911	183	233846	2	*P. ×bretschneideri*	Unknown
X2_snp936	183	530882	2	*P. ×bretschneideri*	Unknown
X3_snp366	115	494820	3	*P. ×bretschneideri*	Unknown
X6_snp1827	67	516082	6	*P. ×bretschneideri*	Unknown
X6_snp1580	506	169138	6	*P. ×bretschneideri*	Unknown
X6_snp1608	506	217289	6	*P. ×bretschneideri*	Pbr029838.1 (unknown)
X6_snp1618	506	217327	6	*P. ×bretschneideri*	Pbr029838.1 (unknown)
X6_snp2558	55014	724	6	*P. communis*	Unknown
X10_snp1622	667	202140	10	*P. ×bretschneideri*	Pbr035011.1 (protein.systhesis.ribosomal)
X10_snp1628	667	202177	10	*P. ×bretschneideri*	Pbr035011.1 (protein.systhesis.ribosomal)
X10_snp1639	667	213771	10	*P. ×bretschneideri*	Unknown
X10_snp1667	667	235820	10	*P. ×bretschneideri*	Pbr035014.1 (unknown)
X12_snp1877	365	232149	12	*P. ×bretschneideri*	Unknown
X12_snp2084	500	245042	12	*P. ×bretschneideri*	Unknown
X14_snp1535	53856	570	14	*P. communis*	Unknown
X15_snp125	111	401240	15	*P. ×bretschneideri*	Unknown
X16_snp57	102	161133	16	*P. ×bretschneideri*	Unknown
X16_snp67	102	161284	16	*P. ×bretschneideri*	Unknown
X16_snp275	185	371007	16	*P. ×bretschneideri*	Pbr011833.1 (RNA.regulation of transcription.PHD finger transcription factor)
X16_snp283	185	371113	16	*P. ×bretschneideri*	Pbr011833.1 (RNA.regulation of transcription.PHD finger transcription factor)

**Table 3:** Single-nucleotide polymorphic (SNP) markers with the largest effect on various traits

*Trait*	*Heritability (%)*	*SNP location*			*Minor allele frequency*			*Significance*		
		*Linkage group*	*Scaffold*	*Position (basepair)*	*Asian*	*European*	*Hybrid*	P*-value*	*FDR*	R^*2*^ *(%)*
FF	67	16	S71.0	485233	0	0.1	0.03	4×10^−7^	0.006	8
TA	17	2	S895.0	79244	0.13	0	0.11	1×10^−5^	0.165	8
Shape	58	13	S83.0	565142	0.09	0.41	0.13	1×10^−5^	0.197	6
RUS	54	8	S398.0	140201	0.22	0	0.03	1×10^−6^	0.021	10
SCU	61	15	S149.0	728797	0.17	0	0.02	8×10^−6^	0.098	9
OCOL	86	9	S155.0	757109	0	0.07	0.02	2×10^−5^	0.172	6

Abbreviations: FDR, false discovery rate; FF, fruit firmness; MAF, minor allele frequency; OCOL, skin over-colour; RUS, skin russet cover; SCU, scuffing; TA, titratable acidity.

Genomic location, MAF in different genetic groups, probability of significance (*P*-value), FDR and percent variance explained (*R*^2^) are shown for each SNP.
