# Supplementary material for: Genotyping-by-sequencing of pear (Pyrus spp.) accessions unravels novel patterns of genetic diversity and selection footprints
Source: Hortic Res. 2017 Apr 12;4:17015–. doi: 10.1038/hortres.2017.15 (PMC5389204; doi:10.1038/hortres.2017.15)
Supplement: Supplementary Information [file hortres201715-s1.docx]

**Genotyping-by-sequencing of pear (*Pyrus* spp.) accessions unravel novel patterns of genetic diversity and selection footprints**

Satish Kumar^1*^, Chris Kirk^2^, Cecilia Deng^3^, Claudia Wiedow^2^, Mareike Knaebel^2^, Lester Brewer^4^

^1^The New Zealand Institute for Plant & Food Research Limited, Hawkes Bay Research Centre, Havelock North, New Zealand.

^2^Palmerston North Research Centre, Palmerston North, New Zealand.

^3^Mount Albert Research Centre, Auckland, New Zealand.

^4^Motueka Research Centre, Motueka, New Zealand.

Corresponding author:

Satish Kumar: [satish.kumar@plantandfood.co.nz](mailto:satish.kumar@plantandfood.co.nz)

This supplementary materials file contains four supplementary figures S1-S5, followed by one supplementary table, S1.

**Supplementary Figure S1**. Fruit shape index assessment by assigning a numerical score to different fruit shapes.


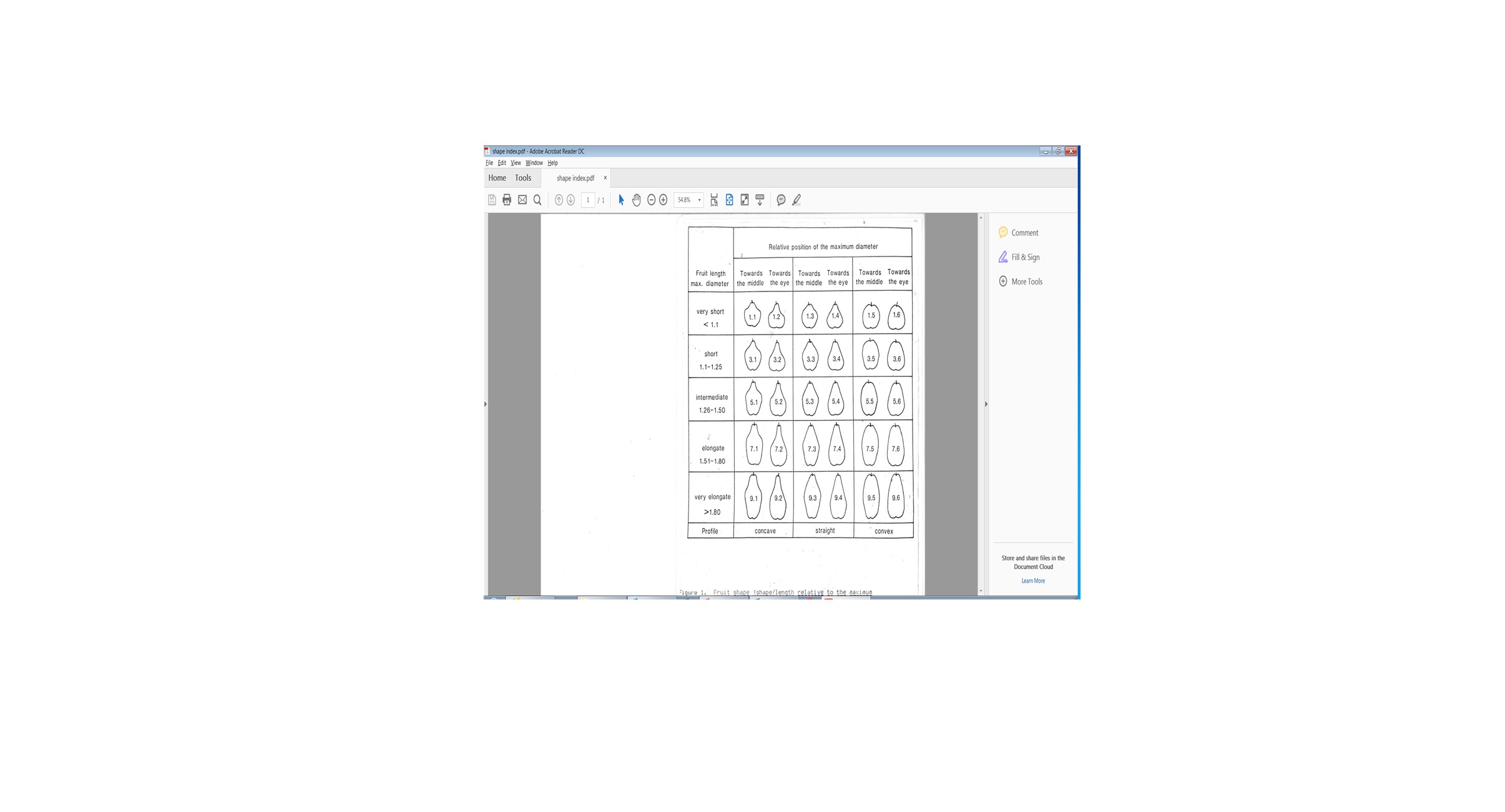


**Supplementary Figure S2**. Genome-wide distribution of population differentiation (*F*_st_) between the three pairs of genetic groups (AS-EU; AS-HY; EU-HY). AS: Asian; EU: European; HY: Hybrid. Scaffold-level averages are shown. Vertical dotted line separates different linkage groups.

1

2

3

4

5

6

8

9

10

11

12

13

14

15

16

17

**Supplementary Figure S3**. Manhattan plots of the -log_10_( p) values for various fruit phenotypes (FF: fruit firmness; TA: titratable acidity; Shape: Shape index; RUS: skin russet cover; SCU: scuffing; COL: over-colour coverage; BIT: skin bitterness) from a genome-wide scan against position on each of 17 linkage groups (represented by different colours).


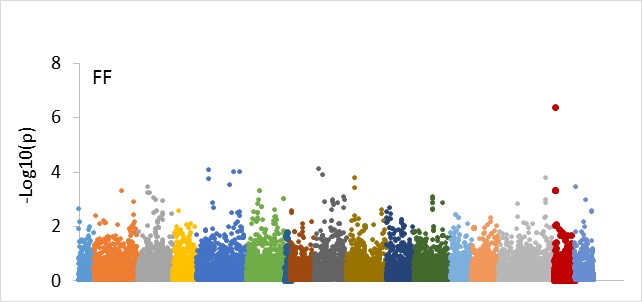

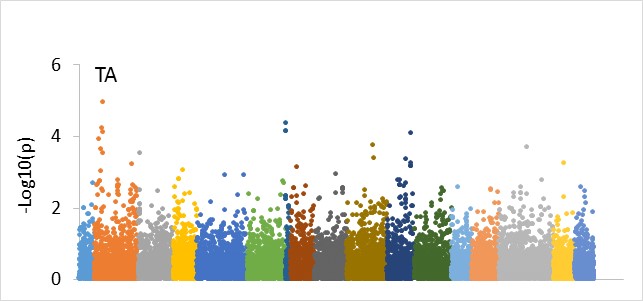

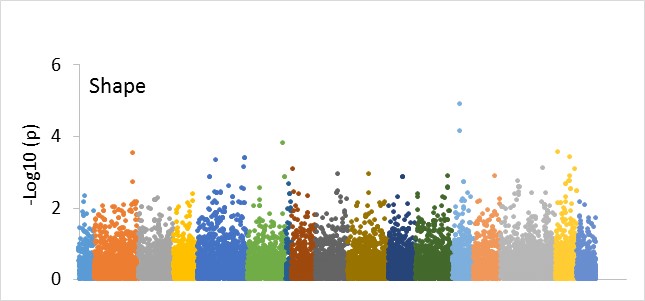

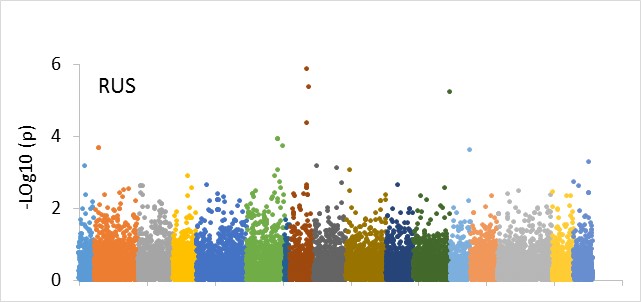

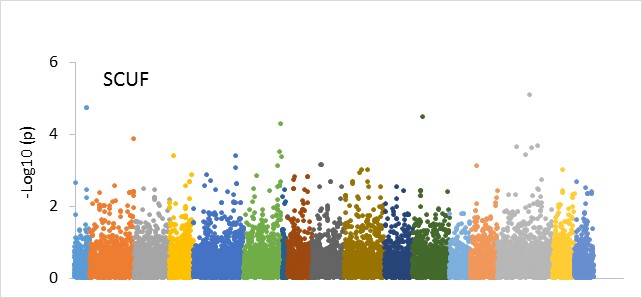

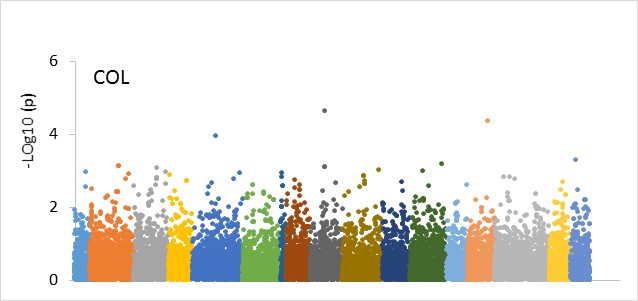

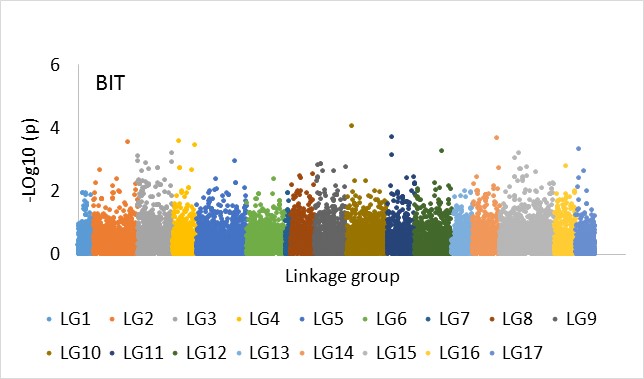


**Supplementary Figure S4**. Quantile-quantile plots for various fruit phenotypes (FF: fruit firmness; TA: titratable acidity; Shape; RUS: skin russet cover; SCU: scuffing; COL: over-colour coverage; BIT: skin bitterness) from a genome-wide scan

BIT

COL

SCU

RUS

Shape

TA

FF

**Supplementary Figure S5**. Flanking sequences of the largest-effect SNP loci listed in Table 3.

S398.0_140201 ATAGGATTTAGTTTCTTTTTGATGTCCCCATGGATTATTTGTTTAGGGTAAACGAATTTTTCTTCAGTGAGATTGGCTATTTCTTTGCTTCTTGATGGATAATGGGTTTAGGTCTTACCAGTTAAATACAATCATGCACGTGTTGTTTTCATACGGCGGATAAATTAGACGGACAAGGATCCTCTTCGGATTCTCTTTGT[C/T]AGGGGATCCAGAAGATTAATCAATCGTGTTTGTTCATCGTACATCGTGCAGTTTGTTTTCATTAGGTACTATTTATATTCAATTTTAAATATAAAATTTAAAATAATTTCGAACCGCACGATATACGACGAACAGACACAATTGATTGGTCCCCGAATTCCCACAAAGAGGATCTGGAGAGGATCCTACTCCAAATTAGA

S155.0_757109 TCAATCGAGTCCTCCCTTCCAGTGTAGAGTTGTAAGCCAAGCTTCTGTTTTGTCTTTGCCTGGAGGGGGTGTCGAGGATCCTCCTTGTAAGAGGGCTAGGCCTAGGTTGGTTCCAATTAGGTATTTCGGCCTGATGTTCAACCTTCAACTTGTTTACTTCCTCAAGGAGTTGTAGGACAAAGGGGTCCTGAGTGGAGTTA[G/A]GTACTGCTGGATCCTTCTTCCGTAAGTCTCCATCTCCTCTTGGAAGTAGGAAAACTTGGTCAAGGGCATGAGATTTTTCTTTAGACTCACCATACTGACTTCCAGGACGAGCCTGTCGAAACGCCCCCGAGTCCCCCGTACCTTCATATTCCTCTAAGACATGTCGCTCATTCCCTAGATTGGCAATTGGCTTGGGGCAT

S71.0_485233 AGGATCGAGCTTTTGGCTGATGCCATTGATCCGAACTTAAGTACTTGGGTACATACTATTCTATCGTATTTGCATCTTATGCTACTACTAGACTGAATATGAATCATTGTTTAAGATGCTGCATCTAAAAATGTGCAATCCGGATTCTTGAATATGTGATTTTTGCATGAATTTCAGATTACGGAAAAGAAGTACATTTG[C/T]TTGTATGGCGGAGAAGACATAGAGTGGATCCGAAACTTCACGAAGGCTGCAAGAAACGTGGCTGTTGAAGCCAACATTCAGTTGGAAATGCTATATGTGGGGCGGAGCAAGCCTAAGGAGACCGTGGTGAAGAACATCATGAACATCATCCAAGCTGAGAAGCTAAGCCACATCCTTGACTTGAACCTCATTTGGTTCTT

S149.0_728797 GTGTCTATTGGGCGTTTTAAGTAGACACTGCTAGCTTTAATTGAAAAAAGGGGTTTTTTTTTTTTTTTTTTACAAAAAGTGGGGGACTTTGGTTTCAAGTCGGGTCCATTTTGTTTTAAAACTAGGTCCACCATTCTTTTAGATAACACATAAGCTTAGACTCAGGAGGATCCTCAATGCTCCTATGGATCATTTAATCA[C/T]ATATGTTCATCGTATATTGTGAGGTCAGAAATTATTGAAATTTTTTTATTTAAAATTAAATATAAATAGTACCTGACAAAAACTGACCGAACGATATACGATAAACGAATATGATTGGAAGATCTCCAGATCCTCACAAAATGGATCCGGCAAGGATCCTTTCTCAGCTTAGACAACCTTGCAAAACTAATAAAAAGAGT

S83.0_565142 TAACAAATCTTTGTCGTGATAGATCGATCCGTTCTTTATTCATTAATTAATCATTGATTTGAAAGTATTAATATGCGTAAAGTTGGCATCTCCTGCGTAATCTATTGATTATAGATTTTAGGTCTCCAAACATGAGAGAGGATCCTCGCTAGATTCTCTTTGTGATGAGGATTCTGGGGATCCTCCAGTCACATCCGTTC[A/G]TCGTACATCGTGCGGTCAATTTTCGTCAGGTACTGTTTATATTCAATTTTAAATAAAAAAATTTACAATGATTTATGACCGCACGATGTACGATGAACATATGTGATTTAAGGATCCCCGAAAATCTCACAAAGAGGATCCGTCGAGGATCCTCACTCTCCAAACATATCTGGAGTGGCCAAAGTAAAGTTTAATTAGGT

S895.0_79244 TTTCAATTGACAAATTATCTATATATATAGGGTGTCTCTACATTTTCATTTAACCTCCCTTCTCCATCACCAACCAATAAAAAACACACATTACATCAAACAACTAGTTATTGGACCATATTTTAAATGGATTTGGATCCCATCTAGATCTAAATTGTGGGAATCGTAGGGATCCTCACATCTTAACCATTCTTCGTGCA[T/C]CGTGGTGTCAGAACATTTGACTTTTTTATTTAAAATTAAACACAAATAGTACCTGACGAAAACTGACCGCACGATATACGATGAACGGTTAGAATGTGAGGATCCCTAAGATTCCGCCAAAGTGGATCCATAAAGGATCCTCTTCCATTTTCAATTACTTCTAGAGTAGTGTAACATAGCCCTATAGTCTAATCTACATT

**Supplementary Table S1**. List of accessions and their *Pyrus* species group.

| CULTIVAR / Selection | Species | CULTIVAR / Selection | Species |
| --- | --- | --- | --- |
| Cangxili | *P. pyrifolia* | *P. calleryana* 2 | *P. calleryana* |
| CAPTAIN HARDY | *P. pyrifolia* | *Pyrus pashia* 1 | *P. pashia* |
| CHIKUSUI | *P. pyrifolia* | 3189 | *P. communis* |
| Choju | *P. pyrifolia* | 2-301 | *P. communis* |
| CHOJURO | *P. pyrifolia* | 6/23/94 | *P. communis* |
| DAN BAE | *P. pyrifolia* | 6-31-100 | *P. communis* |
| DAN BAE | *P. pyrifolia* | 6-31-68 | *P. communis* |
| DOITSU | *P. pyrifolia* | ANGELYS | *P. communis* |
| GION | *P. pyrifolia* | Aurora | *P. communis* |
| Gold Nijisseiki | *P. pyrifolia* | AUTUMN BERGAM | *P. communis* |
| HAKKO | *P. pyrifolia* | BEURRE BOSC | *P. communis* |
| HEISHI | *P. pyrifolia* | BEURRE CAPIAMONT | *P. communis* |
| HOKUSEI | *P. pyrifolia* | BEURRE EASTER | *P. communis* |
| Hougetsu | *P. pyrifolia* | BEURRE HARDY | *P. communis* |
| IMAMURA AKI | *P. pyrifolia* | BEURRE HARDY | *P. communis* |
| NIITAKA | *P. pyrifolia* | BROWN BEURRE | *P. communis* |
| P01 | *P. pyrifolia* | BUTIRRA PRECOCE MORRETINI | *P. communis* |
| P02 | *P. pyrifolia* | BUTIRRA ROSATA MORRETINI | *P. communis* |
| P03 | *P. pyrifolia* | CALIFORNIA | *P. communis* |
| P04 | *P. pyrifolia* | CARMEN | *P. communis* |
| P05 | *P. pyrifolia* | CASCADE | *P. communis* |
| P06 | *P. pyrifolia* | COLETTE | *P. communis* |
| P07 | *P. pyrifolia* | CONCORDE | *P. communis* |
| P08 | *P. pyrifolia* | CRIMSON GEM COMICE | *P. communis* |
| P09 | *P. pyrifolia* | D'Incontinue | *P. communis* |
| P10 | *P. pyrifolia* | DOYENNE DU COMICE | *P. communis* |
| P11 | *P. pyrifolia* | ELDORADO | *P. communis* |
| SEIGYOKI | *P. pyrifolia* | Elizabeth Cole | *P. communis* |
| Shingo | *P. pyrifolia* | FERTILITY | *P. communis* |
| SHINKO | *P. pyrifolia* | FLEMISH BEAUTY | *P. communis* |
| SHINSUI | *P. pyrifolia* | FLORIDA HOME | *P. communis* |
| SUISEI | *P. pyrifolia* | Gleau Morceau | *P. communis* |
| TAMA | *P. pyrifolia* | GOLDEN RUSSET BOSC | *P. communis* |
| WASEAKA | *P. pyrifolia* | GORHAM | *P. communis* |
| Yasato | *P. pyrifolia* | GRAND CHAMPION | *P. communis* |
| B01 | *P. bretschneideri* | HARROW DELIGHT | *P. communis* |
| B02 | *P. bretschneideri* | HIGHLAND | *P. communis* |
| B03 | *P. bretschneideri* | HOWELL | *P. communis* |
| PINGGUOLI | *P. bretschneideri* | HW606 (Harovin Sundown) | *P. communis* |
| QIYUESU | *P. bretschneideri* | JUMBO (STARKS) | *P. communis* |
| TSULI | *P. bretschneideri* | Jupp | *P. communis* |
| XINYALI | *P. bretschneideri* | LOUISE BON DE JERSEY | *P. communis* |
| XUEHUALI | *P. bretschneideri* | MADAME BALLET | *P. communis* |
| YALI | *P. bretschneideri* | Margeurite Marrilat | *P. communis* |
| *P. betulaefolia* 1 | *P. betulaefolia* | MAX RED BARTLETT | *P. communis* |
| *P. calleryana* 1 | *P. calleryana* | MERTON PRIDE | *P. communis* |

(contd)

| CULTIVAR / Selection | Species | CULTIVAR / Selection | Species |
| --- | --- | --- | --- |
| Moders | *P. communis* | RED SENSATION BARTLETT | *P. communis* |
| MOONGLOW | *P. communis* | REIMER RED | *P. communis* |
| Nellie | *P. communis* | ROGUE RED | *P. communis* |
| NEW YORK | *P. communis* | ROSEMARIE | *P. communis* |
| NOUVEAU POITER | *P. communis* | Ruby | *P. communis* |
| OLD HOME | *P. communis* | RX359 | *P. communis* |
| OTTAWA-291 | *P. communis* | RX529 | *P. communis* |
| OVID | *P. communis* | RX810 | *P. communis* |
| C01 | *P. communis* | SIERRA | *P. communis* |
| C02 | *P. communis* | SILVERBELL | *P. communis* |
| C03 | *P. communis* | STARKING DELICIOUS | *P. communis* |
| C04 | *P. communis* | STARKRIMSON | *P. communis* |
| C05 | *P. communis* | SUPER COMICE | *P. communis* |
| C06 | *P. communis* | SWISS BARTLETT | *P. communis* |
| C07 | *P. communis* | TAYLORS GOLD | *P. communis* |
| C08 | *P. communis* | TENN | *P. communis* |
| C09 | *P. communis* | TN09-46 | *P. communis* |
| C10 | *P. communis* | TOSCA | *P. communis* |
| C11 | *P. communis* | US307 | *P. communis* |
| C12 | *P. communis* | US56112/46 | *P. communis* |
| C13 | *P. communis* | UVEDALES ST GERMAINE | *P. communis* |
| C14 | *P. communis* | Velvetine | *P. communis* |
| C15 | *P. communis* | WINTER NELIS | *P. communis* |
| C16 | *P. communis* | WORDEN SECKLE | *P. communis* |
| C17 | *P. communis* | Crispie | Hybrid |
| C18 | *P. communis* | HWA HONG | Hybrid |
| C19 | *P. communis* | MAXIE | Hybrid |
| C20 | *P. communis* | H01 | Hybrid |
| C21 | *P. communis* | H02 | Hybrid |
| C22 | *P. communis* | H03 | Hybrid |
| C23 | *P. communis* | H04 | Hybrid |
| C24 | *P. communis* | H05 | Hybrid |
| C25 | *P. communis* | H06 | Hybrid |
| C26 | *P. communis* | H07 | Hybrid |
| C27 | *P. communis* | H08 | Hybrid |
| P327-57 | *P. communis* | H09 | Hybrid |
| PACKHAM'S TRIUMPH | *P. communis* | H10 | Hybrid |
| PASSA CRASSANA | *P. communis* | H11 | Hybrid |
| PATTEN | *P. communis* | H12 | Hybrid |
| Peamy | *P. communis* | H13 | Hybrid |
| PIERRE CORNEILLE | *P. communis* | H14 | Hybrid |
| PRESIDENT D'OSMOND | *P. communis* | H15 | Hybrid |
| President Heron | *P. communis* | H16 | Hybrid |
| PRINCESS | *P. communis* | H17 | Hybrid |
| PT AV-63-2076 | *P. communis* | H18 | Hybrid |
| RED ANJOU | *P. communis* | H19 | Hybrid |

(contd)

| CULTIVAR / Selection | Species |
| --- | --- |
| H20 | Hybrid |
| H21 | Hybrid |
| H22 | Hybrid |
| H23 | Hybrid |
| H24 | Hybrid |
| H25 | Hybrid |
| H26 | Hybrid |
| H27 | Hybrid |
| H28 | Hybrid |
| H29 | Hybrid |
| H30 | Hybrid |
| H31 | Hybrid |
| H32 | Hybrid |
| H33 | Hybrid |
| H34 | Hybrid |
| H35 | Hybrid |
| H36 | Hybrid |
| H37 | Hybrid |
| H38 | Hybrid |
| H39 | Hybrid |
| H40 | Hybrid |
| H41 | Hybrid |
| H42 | Hybrid |
| H43 | Hybrid |
| H44 | Hybrid |
| H45 | Hybrid |
| H46 | Hybrid |
| H47 | Hybrid |
| H48 | Hybrid |
| H49 | Hybrid |
| H50 | Hybrid |
| H51 | Hybrid |
